# Liver Transplantation in a Patient With Hepatic Angiosarcoma

**DOI:** 10.7759/cureus.12609

**Published:** 2021-01-10

**Authors:** Amaninder Dhaliwal, Annie Braseth, Banreet S Dhindsa, Daryl Ramai, Fedja A Rochling

**Affiliations:** 1 Gastroenterology and Hepatology, University of South Florida Morsani College of Medicine, Tampa, USA; 2 Gastroenterology and Hepatology, The University of Iowa Carver College of Medicine, Iowa City, USA; 3 Gastroenterology and Hepatology, University of Nebraska Medical Center, Omaha, USA; 4 Internal Medicine, The Brooklyn Hospital Center, New York, USA

**Keywords:** primary angiosarcoma, liver transplantation, benign vascular tumor, case report

## Abstract

Liver transplantation (LT) is an accepted form of therapy for selected cases of malignant tumors of the liver that include primary and fibrolamellar hepatocellular carcinoma, cholangiocarcinoma limited to Klatskin distribution, neuroendocrine tumors, epithelioid hemangioendothelioma, and hepatoblastoma. This is the case of a 61-year-old previously healthy female transferred from an outside hospital for a second opinion for a liver transplant. Computed tomography of the abdomen with contrast showed cirrhosis and multiple masses with arterial enhancement in her liver. She underwent a liver biopsy that showed a low-grade vascular tumor. She underwent an exploratory laparotomy with open liver biopsy which showed no visual evidence of omental spread. The pathology was reported as a low-grade vascular lesion, which was likely a small vessel neoplasm. After denial for LT secondary to an unknown low-grade vascular tumor, she presented to our medical center. Oncology was consulted and diagnosed with her liver vascular tumors as benign with an overall favorable prognosis. She was listed for liver transplant with a model for end-stage liver disease-sodium score of 25 and developed hepatorenal syndrome type 1. She was on hemodialysis for approximately 10 weeks prior to her LT and was eventually listed for simultaneous liver and kidney transplants. She underwent an orthotopic liver transplant 10 weeks after presenting to UNMC. The amount of necrosis and the elevated mitotic rate was sufficient to classify the tumor as a Federation Nationale des Centres de Lutte le Cancer grade 3 of three angiosarcomas. She was scheduled for a living donor kidney transplant three days after her liver transplant, but it was postponed after she continued to have increased urine output that responded to a trial of diuretics with continued improvement in kidney function. She successfully completed 16 months post-LT without any known recurrence of primary angiosarcoma.

## Introduction

Liver transplantation (LT) has evolved as a treatment for hepatic malignancies with favorable survival for those with primary hepatocellular carcinoma (HCC) without metastatic disease [1]. The use of transplantation for the management of primary angiosarcoma of the liver remains controversial given the poor survival rates with almost all recurring within the first six months after transplant [2]. These tumors originate from hepatic endothelium and aggressively fill the hepatic sinusoids leading to hepatic atrophy [1,3].^ ^When looking at primary hepatic cancers, these tumors only represent 0.1-2% of the cases.

## Case presentation

**Figure 1:**
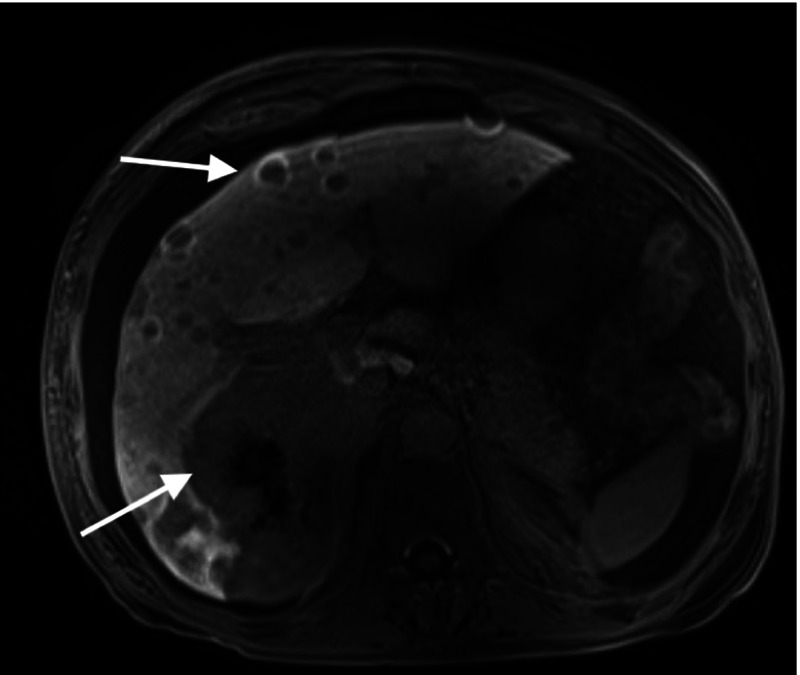
MRI of the liver prior to transplant. Arrows are depicting multiple enhancing liver masses.

A 61-year-old previously healthy female was transferred from an outside hospital for a second opinion for a liver transplant. She first noticed fatigue, weakness, and abdominal swelling three months prior to her admission at the outside hospital. CT of the abdomen with contrast showed cirrhosis and multiple masses with arterial enhancement in her liver. She underwent a liver biopsy. Pathology was reported as a low-grade vascular tumor. She presented to a larger academic center where she underwent an exploratory laparotomy with open liver biopsy to further investigate the liver lesions. There was no visual evidence of omental spread. The pathology was reviewed by multiple tertiary centers specializing in hepatic malignancies and was reported as a low-grade vascular lesion, which was likely small vessel neoplasm. After denial for LT secondary to an unknown low-grade vascular tumor, she presented to our medical center. MRI of the liver showed multiple enhancing liver masses (Figure 1). Oncology was consulted and she was diagnosed with benign liver vascular tumors with an overall favorable prognosis.

She was listed for liver transplant with a model for end-stage liver disease-sodium (MELD-Na) score of 25. The patient had normal renal function prior to her admission with a creatinine level of 0.79 mg/dL, which increased to 3.3 mg/dL after her admission which was thought to be secondary to hepatorenal syndrome (HRS) type I. Her creatinine level did not improve despite medical management and hemodialysis (HD) was initiated prior to transfer to the University Medical Center. She continued on HD for approximately 10 weeks prior to her LT and was listed for combined liver and kidney transplant.

**Figure 2:**
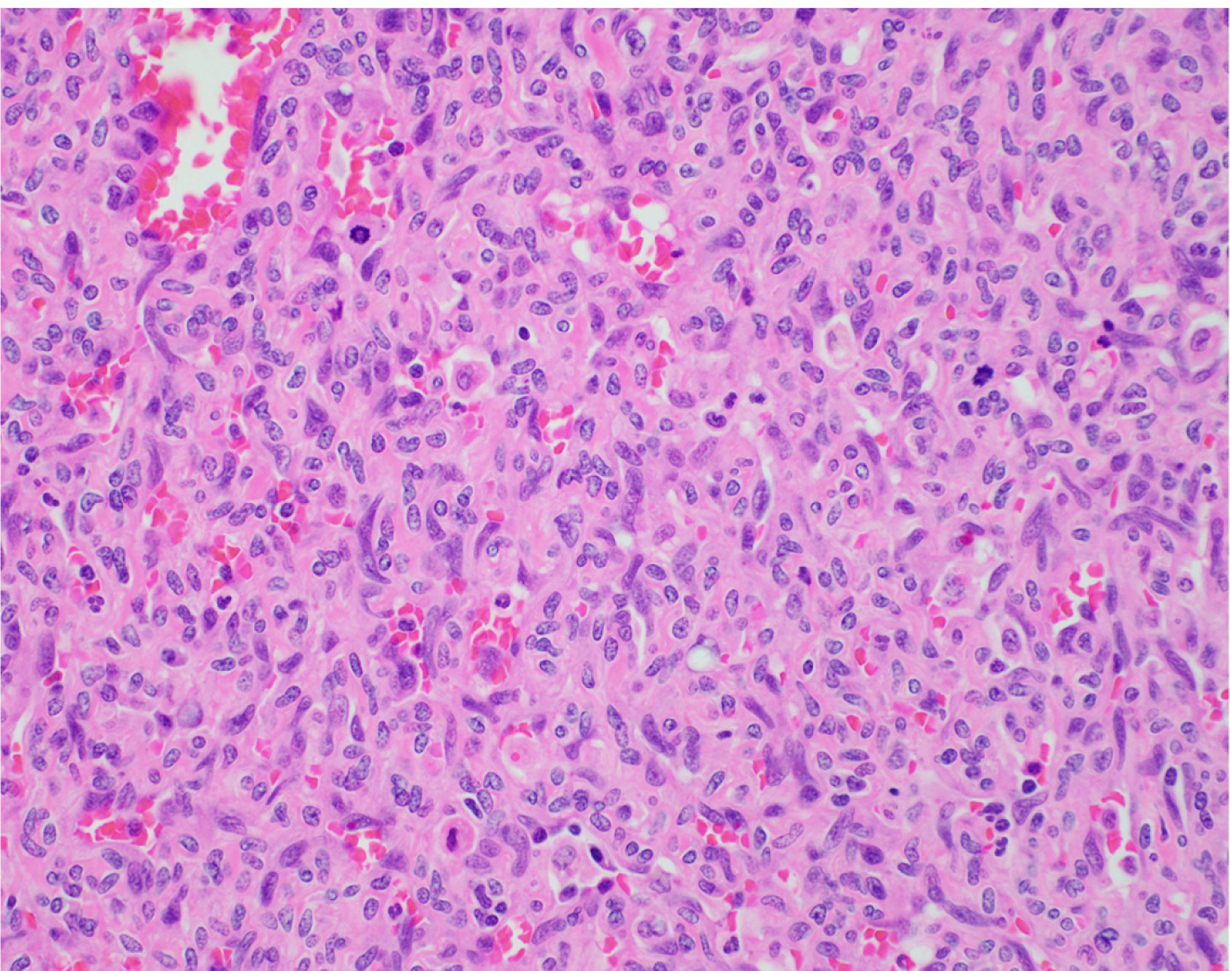
High-power view of the tumor showing solid areas of highly packed atypical cells with occasional vascular channels and numerous atypical mitotic figures.

She underwent an orthotopic liver transplant 10 weeks after presenting to the UNMC. Microscopic evaluation of the tumor cells showed positive immunohistochemical staining for the ETS-related gene and was negative for human herpesvirus-8. A Ki-67 labeling index of the solid areas was approximately 30%. Overall, the amount of necrosis and the elevated mitotic rate was sufficient to classify the tumor as a Federation Nationale des Centres de Lutte le Cancer grade 3 of three angiosarcomas (Figure 2).

She was scheduled for a living donor kidney transplant three days after her liver transplant, but it was postponed due to increased urine output on postoperative day four. Her creatinine level was 2.22 mg/dL and blood urea nitrogen was 73 mg/dL at the time of discharge.

She was undergoing routine image testing every three months after transplantation. Unfortunately, she was found to have a recurrence in the form of innumerable liver and bone lesions at 16 months post-transplantation. Despite starting her on chemotherapy with taxol, the patient passed away 31 months post-LT.

## Discussion

Primary angiosarcoma usually present in the sixth to seventh decades of life with a 3:1 male-to-female predominance [3,4]. Environmental carcinogens, especially vinyl chloride, are associated with an increased risk of developing hepatic angiosarcoma; however, in most cases, the cause remains unknown [5]. Lesions seen on CT and MRI imaging can show increased vascularity, while others can be hypodense or even appear normal [6,7]. The overall prognosis is very poor, with an average survival of less than six months from the time of diagnosis [5].^ ^There is currently no standard treatment, but survival benefit has been shown in examples of local excision, with or without adjuvant therapy. However, less than 20% of patients will have resectable disease at the time of diagnosis [5]. The tumor does not respond to radiation therapy and no standard chemotherapy exists [7].^ ^The European transplant registry lists primary hepatic angiosarcoma as an absolute contraindication to transplant [8].

Our patient presented with weakness, fatigue, and abdominal swelling. Imaging showed multiple enhancing liver masses and her biopsy was reported as a low-grade vascular lesion by three, separate, highly respected institutions. Pretransplant tumor diagnosis was unknown, and the diagnosis was made after she was transplanted.

Husted et al. examined six patients at three different transplant centers who all underwent liver transplants for hepatic angiosarcomas. All six patients had no evidence of metastatic disease at the time of transplant. Unfortunately, all patients had recurrent disease after transplant with a median interval of recurrence of two months, with only one patient surviving beyond one year [2]. Gonzalez et al. identified one patient with angiosarcoma who underwent a liver transplant. The patient died five months after the transplant due to tumor recurrence [1]. Yoshida et al. reported two patients with primary hepatic angiosarcoma who were diagnosed with a histopathologic examination after transplantation. In both cases, imaging was nonspecific and biopsy was not done. One patient had recurrence after nine months and passed away 27 months after the transplant, while the other had immediate tumor recurrence and passed away six months after transplant [9]. A literature review looking at a total of 22 patients who underwent liver transplants found that median survival was six months, with 77% of the patients dying of tumor recurrence [5]. Orlando et al. conducted a retrospective review of 14 males and eight females reported to the European Liver Transplant Registry who were transplanted for angiosarcoma. All patients died before the end of the second post-transplantation year. The longest survival was 23 months in a patient with primary angiosarcoma [10].

## Conclusions

To our knowledge, this is a unique case, with the longest survival (31 months) after LT in a patient with primary angiosarcoma. The decision to transplant was not taken easily and came about after denials at other transplant centers. With the current allocation and the number of recipients awaiting liver transplants, the ethics of transplanting for such an aggressive and lethal malignancy remains controversial.
